# Potential role of lncRNA cyp2c91–protein interactions on diseases of the immune system

**DOI:** 10.3389/fgene.2015.00255

**Published:** 2015-07-28

**Authors:** Prashanth Suravajhala, Lisette J. A. Kogelman, Gianluca Mazzoni, Haja N. Kadarmideen

**Affiliations:** Department of Veterinary Clinical and Animal Sciences, Faculty of Health and Medical Sciences, University of Copenhagen, FrederiksbergDenmark

**Keywords:** long non-coding RNA, systems biology, protein interactions, disease association, WGCNA

## Abstract

With unprecedented increase in next generation sequencing technologies, there has been a persistent interest on transcript profiles of long non-coding RNAs (lncRNAs) and protein-coding genes forming an interaction network. Apart from protein–protein interaction (PPI), gene network models such as Weighted Gene Co-expression Network Analysis (WGCNA) are used to functionally annotate lncRNAs in identifying their potential disease associations. To address this, studies have led to characterizing transcript structures and understanding expression profiles mediating regulatory roles. In the current exploratory analysis, we show how a lncRNA – cyp2c91 contributes to the transcriptional regulation localized to cytoplasm thereby making refractory environment for transcription. By applying network methods and pathway analyses on genes related to a disease such as obesity and systemic lupus erythematosus, we show that we can gain deeper insight in biological processes such as the perturbances in immune system, and get a better understanding of the systems biology of diseases.

## Introduction

The long non-coding RNAs (lncRNAs) are known to be involved in several biological roles including epigenetic regulation, immune signaling, aberrant methylation of imprinted genes and cell cycle (Li et al., 2015). Distinct lncRNA expression profiles are associated with recurrent mutations linked to cancer and therapy related diseases (Garzon et al., 2014). With unprecedented increase in next generation sequencing (NGS) technologies, there has been a persistent interest on transcript profiles of lncRNAs and protein-coding genes forming an interaction network. Apart from protein–protein interaction (PPI), co-expression models such as Weighted Gene Co-expression Network Analysis (WGCNA; Xue et al., 2013) are used to functionally annotate lncRNAs in identifying their potential disease associations (Cogill and Wang, 2014). To address this, studies have led to characterizing transcript structures and understanding expression profiles mediating regulatory roles and comparing them with the ENCODE project (ENCODE Project Consortium, 2004). Recent reports show how lncRNAs contribute toward regulatory interactions with their non-coding peers like miRNAs (Jalali et al., 2013). It is not well-known whether lncRNA-protein networks restrain interactions. How such regulatory interactions between classes of lncRNAs and proteins would have a significant influence on the organism remains a challenge.

**FIGURE 1:**
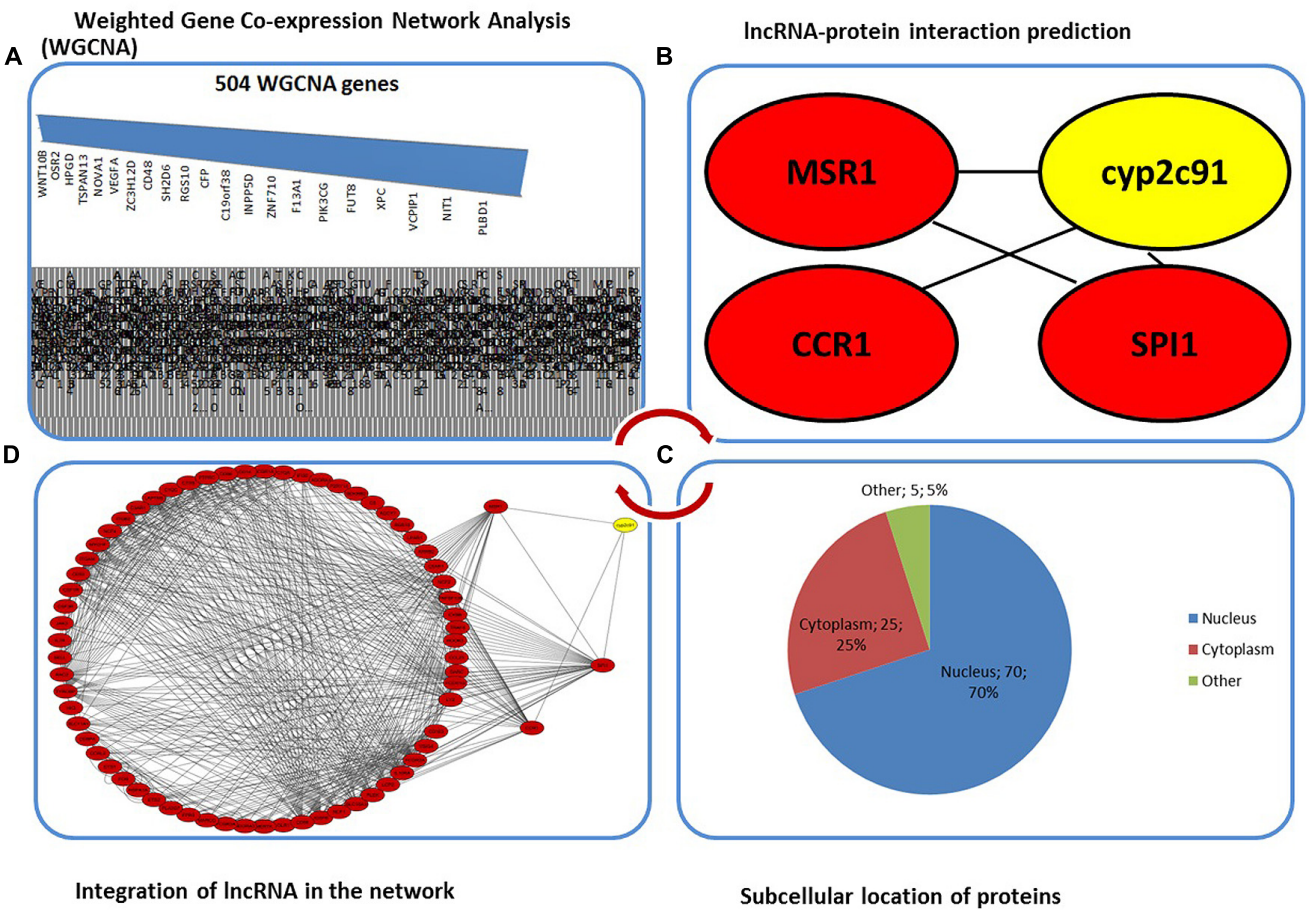
**(A)** The 504 genes from WGCNA across different modules linked to diseases not limited to obesity and immune response. **(B)** Representative lncRNA cyp2c91 gene (in yellow) shown to be interacting with three regulatory protein-coding genes predicted from RPI-pred. **(C)** Subcellular location of the genes associated in the network analyzed from TargetP and Cello predictions (Emanuelsson et al., 2000; Yu et al., 2014). **(D)** The network topology showing the profiled expression across the regulatory genes associated with cyp2c91. This is indicative of global protein-RNA interaction data.

Earlier, we have shown three regulatory genes, *viz.* chemokine (C–C motif) receptor 1 (*CCR1)*, macrophage scavenger receptor 1 (*MSR1)* and spleen focus forming virus proviral integration oncogene (*SPI1)* associated with diseases like obesity and osteoporosis using gene network algorithms WGCNA and Lemon-Tree (Kogelman et al., 2014) applied to NGS-based RNA-Seq datasets from porcine model for obesity (*see RNA-Seq web reference*^1^). These clusters of highly co-expressed genes were ranked as highly significant based on their association with obesity-related phenotypes. With a wide range of biological processes effectively used as regulatory molecules, we anticipate (a) if the co-expressed genes have interacting partners with any lncRNAs, (b) if so, whether they affect the co-expression, further changing the networks and influencing the organismal phenotype or disease outcomes, or (c) if not, what would be the outcome of such lncRNA-dependent transcription. From a putative interaction network visualized using Cytoscape (Lopes et al., 2010), we have established functional classes based on several different methods, explicitly focusing on the betweenness centrality, closeness centrality and presence of subcellular location signals (see **Figure 1**). These resilient methods would distinguish probability of lncRNA to show association/disassociation paradigm, RNA binding protein-lncRNA interactivity and importantly disease association, if any.

## Computational Methods for lncRNA cyp2c91–Protein Interaction

In the current study, we made a human concordant network after previously published WGCNA result from an animal model (Kogelman et al., 2014) and found that 340 of 504 porcine genes have ortholog peers in humans (**Figure 1A**). The 504 genes were extracted from a summated list of different modules containing clusters of highly co-expressed genes (see Supplementary Table S2). The absence of orthologs in human is in agreement with the homology data available from the Pig Analysis Database (PAD), which specifies that about 73% of the sequences are covered by the both genomes (*see PAD web reference*^2^). With the hypothesis that lncRNA–protein interactions play an important role in regulating post-transcriptional changes and subsequent localization of the transcript, we used RNA–protein interaction predictor (RPI-pred) to predict whether or not the proteins encoded by these genes and the RNA form interaction pairs (Suresh et al., 2015). From these networks and GenBank annotation, we observed that *cyp2c91*, a lncRNA interacts with a host of regulatory genes. The betweenness and closeness centralities were computed using Cytoscape. The topological parameters containing betweenness and closeness centralities were taken from a host of parameters (average clustering coefficient, betweenness centrality, closeness centrality, neighborhood connectivity, node degree distribution, shared neighbors, shortest length, stress centrality, topological coefficients) that are ideally calculated by Cytoscape by default. Computing these centrality indices would accomplish identifying the relationship between the nodes, understanding node-by-node quantification (Su et al., 2014). Furthermore, a classification of such nodes would allow us to understand their capability to influence the function of nodes/genes in the network, where in this case, lncRNA–protein interactions. As the betweenness centrality is computed for the interaction networks that do not contain multiple edges, closeness centrality was also added as an indicative which is reciprocal of the average shortest path length between the nodes (see Supplementary Table S1). The betweenness centrality of *cyp2c91* with the three regulator genes linked to obesity (*CCR1*, *MSR1,* and *SPI1*) was found to be between 0 to 1 (**Figure 1B**). Considering the fact that these small molecules enter the nucleus without regulation, we asked if any gene products are localized to nucleus. From the subcellular prediction tools (Emanuelsson et al., 2000; Yu et al., 2014), we observed that among the three regulator genes, *CCR1* was found to be localized to cytoplasm (**Figure 1C**). Encouraged by the outcome that the three have a plausible role of interaction with *cyp2c91*, we made a reliable interaction network with the mean disassociation based on the betweenness centrality (**Figure 1D**). We found that *MSR1* and *SPI1* form interacting pairs with each other while *CCR1* was a lone gene. Nonetheless, the lncRNA–protein interactions were extended with the *CCR1*-*cyp2c91* association mapped from network genes. The study suggests two ways forward. First, the fold change can be attributed to lncRNA-dependent transcription. Second, *CCR1*-*cyp2c91* association is significant when compared to *MSR1*-*cyp2c91* and *SPI1*-*cyp2c91* where the genes are regulatory in nature forming diseased network. The three regulatory genes are associated with obesity and immune system, possibly linking them to Lupus. This is evident by the fact that several genes present in the WGCNA modules o*f* Kogelman et al. (2014; *TNIP1, GPSM3, TFEC, TES, KCP, IRF5, TNPO3, ELF1, ITGAM and TNXB, KLF6, AKR1E2*) are related to nuclear factor-kappaB (*NF-κB*) signaling pathways classified to immune system and systemic lupus erythematosus (SLE; Cen et al., 2013). This might allow us to use this network as a model for immune response or obesity.

## Conclusion

The genome is lengthily transcribed in eukaryotes and it has been known that many transcripts have larger proportion of non-coding components. Although about 66–73% of the porcine genome (including ESTs, genes etc.) is conserved across humans, a considerable set of genes regulate interactions with lncRNAs. Further, a range of transcribed regions might tend to be regulatory and indicative of enhancing non-functional activity. Moving to a broader spectrum of calling them as junk, we ask for evidences on their regulatory potential based on their association with protein-coding genes. Consistent with the interaction networks, subcellular localization of the products of the three protein-coding genes revealed that two are nuclear while one, *CCR1* was found to be in cytoplasm. This is again in agreement with the fact that the subcellular fractions of lncRNA differ significantly from each other, with a majority enriched in the nucleus, cytoplasm, and ribosomes (van Heesch et al., 2014). These results show that lncRNA–protein interactions are self-regulating and may not be influenced by organellar specificity. Our exploratory analysis suggests that *CCR1-cyp2c91* contributes to the transcriptional regulation localized to cytoplasm thereby making refractory environment for transcription. By applying network methods and pathway analyses on genes related to a disease such as obesity and SLE, we show that we can gain deeper insight in biological processes such as the perturbances in immune system, and get a better understanding of the systems biology of diseases. This stresses the possible need of finding genes linked to lncRNA-protein networks and further use them as potential diagnostic markers.

## Conflict of Interest Statement

The authors declare that the research was conducted in the absence of any commercial or financial relationships that could be construed as a potential conflict of interest.

## Footnotes

1 http://www.ncbi.nlm.nih.gov/geo/query/acc.cgi?acc=GSE61271

2 http://www.piggenome.dk/
