# Supplementary material for: Potential role of lncRNA cyp2c91–protein interactions on diseases of the immune system
Source: Front Genet. 2015 Jul 28;6:255. doi: 10.3389/fgene.2015.00255 (PMC4516971; doi:10.3389/fgene.2015.00255)
Supplement: Supplementary file 1 [file Table_1.DOCX]

Supplementary Table 1:

Supplementary Table 1: The betweenness centrality (BC) and closeness centrality (CC) values for the nodes were computed and visualized using Cytoscape. The topological parameters containing betweenness and closeness centralities were taken from a host of parameters (average clustering coefficient, betweenness centrality (BC), closeness centrality (CC), neighborhood connectivity, node degree distribution, shared neighbors, shortest length, stress centrality, topological coefficients) that are calculated by Cytoscape. Computing these centrality indices would accomplish identifying the relationship between the nodes, understanding node-by-node quantification. The centrality values are computed for those that do not contain multiple edges. They are the normalized values for each gene/node by dividing the number of pairs of nodes existing in the network. The range would be between 0 to 1 with the condensed values in exponential form (centrality of vertex). From the network analyzer, the essentiality of the node in the form of BC was calculated based on the formula
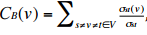
 . The BC for a node v is obtained from this formula where
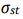
the number of shortest path from s to t is and
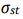
(v) is again the shortest path from s to t via v, which means removal of a node, will bring a significant effect in the network to change. This is evident for those values that are less and those values that are not essentially known. Similarly, CC is a degree to which the node is close to the neighboring nodes and is known from the formula:
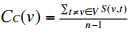
 where S (v, t) is the shortest path between v and t. In our network, the values are appropriate with all the three genes networked. For those nodes that centrality is not calculated, - (dash) is seen as demarcated.
