# Supplementary material for: Potential role of lncRNA cyp2c91–protein interactions on diseases of the immune system
Source: Front Genet. 2015 Jul 28;6:255. doi: 10.3389/fgene.2015.00255 (PMC4516971; doi:10.3389/fgene.2015.00255)
Supplement: Supplementary file 2 [file Table_2.DOC]

Supplementary Table 2: List of 504 WGCNA genes (from Figure 1; Panel a)

WNT10B

MFAP5

BEND6

LOXL1

NPTN

CCDC88C

TPPP3

RBP7

EMILIN2

ROR1

PDE10A

FNDC1

ENPP1

SOBP

FYN

FAM46A

MEX3C

TMOD2

FBN1

NEDD4L

ITGA11

LARP6

LPAR1

COL14A1

OSR2

PDP1

PI15

MSC

DPT

UAP1

THBS3

ADAMTSL4

MAB21L3

FAM102B

TGFBR3

EDEM2

TSHZ2

FOXK1

EEF2K

EFEMP1

VIT

DKK2

PRDM8

ANTXR2

MEDAG

CAB39L

PCDH17

SCARA5

HPGD

GPR133

CAMKK2

MCU

KAT6B

PLAC9

ANXA8

ADD3

NTRK2

PFKP

GALNT15

TGFBR2

ACKR2

CNTN3

CLSTN2

PCOLCE2

PLD1

CHST7

AR

BGN

LRP4

FEM1C

ARHGAP26

ARHGAP32

TSPAN13

C1orf21

PDLIM3

CCDC141

ITGAV

FN1

EFHD1

WDR86

DENND2A

FAM3C

WNT2

ADAMTS12

C1QTNF3

ADAMTS6

ADAM19

ADAMTS16

PLXDC1

SMIM14

PCSK6

CD9

ROPN1L

PLA2G4A

PCSK5

TNXB

NOVA1

SNX24

PROCR

MAMDC2

BDKRB2

SAMHD1

KLF6

GPR64

CST3

CYTH4

CSF2RB

NCF4

LYZ

FGD4

ARHGDIB

OLR1

CLECL1

PLXNC1

SERPINB1

KDM1B

AIF1

GPSM3

DMB

FGD2

VEGFA

NFKBIE

CTSH

BCL2A1

PSTPIP1

CD22

TYROBP

RASGRP4

MAP4K1

TGFB1

PLAUR

BAX

CD33

NKG7

SIGLEC5

CH242-402I11.1

EFHD2

C1QA

C1QC

RPS6KA1

THEMIS2

MYCL

SMAP1L

TAGAP

ZC3H12D

ABRACL

OGFRL1

ME2

PLEKHO2

PLCB2

ATP10A

SERPINB10

SLC38A6

PTPLAD2

GNG10

SLC46A2

C5

PBX3

FAM49B

SDC2

SDCBP

LYN

SELL

RCSD1

CD32

FCER1G

ARHGAP30

LY9

CD48

TPM3

S100A10

CDC42SE1

CTSS

FCGR1A

CTTNBP2NL

CD53

DPYD

GSDMD

MSR1

ACSS1

MAPRE1

AHCY

PTGIS

CTSZ

ARPC1B

LRCH4

NCF1

GUSB

ITGAM

PYCARD

ARID5A

CAPG

SH2D6

DOK1

ARHGAP25

PLEK

PQLC3

RHOH

STAP1

SPP1

ALOX5AP

LCP1

ELF1

KCTD12

TNFSF13B

SLC18A1

DOK2

TAOK3

SLC8B1

AP1B1

EGR2

LIPA

BLNK

PIK3AP1

PI4K2A

KIAA1598

RGS10

GMFG

ADAM8

RGS2

PTPRC

CTSL

CH242-21O2.1

MYD88

CCR1

STAB1

FRMD4B

P2Y12R

NCEH1

CD86

HCLS1

CD200R1

NXPE3

CHODL

ARHGAP6

TLR7

TLR8

TMSB4

SYAP1

CYBB

CFP

IL2RG

SASH3

ZDHHC9

IGSF1

ARHGAP4

TCIRG1

PTPRCAP

FERMT3

CCDC88B

MS4A4A

SLC15A3

MPEG1

FAM111B

SPI1

ACP2

GNA15

DENND1C

VAV1

EMR1

STXBP2

CD209

MYO1F

ACP5

C19orf38

DNASE2

CD97

GLT25D1

JAK3

IFI30

GMIP

NLRP3

HEXB

TNFAIP8

CD14

CSF1R

UCP2

IL10RA

UBASH3B

VWA5A

SCIN

NCF2

ARPC5

RGL1

ATF3

ARP3

ADAM-9

ITGA4

INPP5D

GPC1

GIMAP1

GIMAP8

TBXAS1

IRF5

TFEC

FAM105A

IL7R

FYB

LCP2

DOCK2

TNIP1

CD300C

GRN

INTS2

EVI2B

SCIMP

ARRB2

CD68

PIK3R5

TRPV2

EMR4

LPXN

ZNF710

SOAT1

CD83

RAB31

ADCY7

RAC2

TNFRSF1B

FOLR1

GPR34

CD37

DNASE1L1

AMPD3

SLC37A2

TSHR

PLEKHO1

SRGN

PRKCD

NRROS

CCRL2

CPM

CTSB

PTPRJ

C3AR1

CPPED1

F13A1

CCR2

SELPLG

APOBR

LAPTM5

NRAMP1

ITGB2

FXYD5

PTPN6

FJX1

PCD1A

KMO

CSF3R

MFSD7

MFSD12

CTSD

PADI2

TLR1

HMOX1

CYP2C42

DRAM2

GLA

GIMAP6

SLC35G1

PIK3CG

WIPF1

LAT2

HMHA1

GIMAP4

IGSF6

DAPP1

RARRES1

CASP1

PLOD3

BCAT1

PigE-108A11.6

C5AR1

RHOG

CORO1A

C16orf54

CYTIP

CSF2RA

COTL1

MAFB

ANO6

NTN4

C2

IL-16

FUT8

PLD4

AXL

SMAD2

SERPINB8

ARF6

LGALS3

PTGS1

MAL2

PMVK

S100A4

IKBKB

IL4R

MGAT4A

CALM1

CLU

CMLKR1

SLC29A3

FAT4

UBTD1

VIM

MSRB2

PIP4K2A

NCKIPSD

XPC

P2RY14

COL8A1

AP1S2

MAP7D2

TIMP1

NAP1L3

ELF4

CLIC2

MS4A2

HK3

CAST

DIAPH1

CD55

KYNU

ZAP

CCL23

GAS7

FAM20C

TTC36

PRR5

RBPJ

RHBDF2

EMP3

VCPIP1

CLCN4

TUBB6

PTGIR

OSBPL11

SIRPB1

ECM1

LYVE1

APBB1IP

MAFF

KRR1

LYRM5

ALDH1L2

PRPF4B

C6orf106

AKT2

CHD5

SLC25A34

PINK1

TINAGL1

NEXN

DHRS7

TDRD7

GGTA1

NIT1

DARC

SMG5

PTCD3

MORN2

BTC

P2RX6

NOC3L

AS3MT

FGFR2

AQP7

P2RY1

SMS

MACROD1

CYP2R1

ME3

APLP2

OXCT1

ADORA1

OPTN

NFE2L1

RPL13

PLA2G16

CAMSAP2

PLBD1

DDX51

XBP1

NOB1

TAF4B

AMD1

EXOSC2

RAE1

POLR3E

ABCE1

RANBP1

URB2

NPM3

MANF

GNL3

SSR4

PPP1R14B

EIF4A1

LMNB2

SRM

POLR1C

WDR74

FBXL7

NOL9
